# Dust Deposition Impacts at a Liquidated Gold Mine Village: Gauteng Province in South Africa

**DOI:** 10.3390/ijerph17144929

**Published:** 2020-07-08

**Authors:** Mbalenhle Mpanza, Elhadi Adam, Raeesa Moolla

**Affiliations:** 1 Department of Mining Engineering and Mine Survey, University of Johannesburg, 55 Beit St, Doornfontein 2028, South Africa; 2 School of Geography, Archaeology and Environmental Studies, University of the Witwatersrand, Private Bag X3, Braamfontein 2050, South Africa; Elhadi.Adam@wits.ac.za (E.A.); Raeesa.Moolla@wits.ac.za (R.M.)

**Keywords:** TSFs, windblown dust, respiratory illnesses, mining, community, heavy metals, PM_10_

## Abstract

The windy season brings numerous community complaints for gold mining companies situated in the Witwatersrand due to windblown dust from partially rehabilitated tailings storage facilities (TSFs). For communities encroaching onto TSFs, windblown dust is perceived as a health hazard and an environmental challenge. In a study conducted in 2017 by the Lawyers for Human Rights, the community of a gold mine village perceived tailings storage facility 6 (TSF6) and other surrounding tailings storage facilities which are partially rehabilitated to be a health and socio-economic threat. Since 2013, when a nearby gold mining company was liquidated, this community has been complaining about dust fallout. To validate the claims made by the community this paper reports on the dust deposition impacts, and respiratory illnesses risk posed by wind-blown generated dust. The study conducts an air quality assessment using dispersion modelling of windblown dust. Surface material from the TSFs was sampled, analysed for silica and heavy metal content using X-ray fluorescence (XRF) and inductively coupled plasma-mass spectrometry (ICP-MS) respectively. This study finds that PM_10_ dust fallout, high in silica and uranium content, could potentially pose health threats to the surrounding community. The study further shows that dust deposition is the highest in July–October, with TSF6 posing a nuisance while TSF1 represents a potential health threat owing to its particle size distribution for the surrounding gold mine village community. Potential receptors of the air pollution by dust in this study area include neighbouring property owners, business owners of the nearby shopping centre, the school and the clinic. This study further finds that sudden mine closure due to mine liquidation results in unrehabilitated tailings storage facilities which exacerbates dust deposition.

## 1. Introduction

Deposition dust is the primary particulate material defined by Pöschl [1] as any material which can either be emitted as liquids or solids from natural sources: biogenic materials (pollens, spores, micro-organisms, insects and needle-shaped particles), volcanic eruptions, biomass burning, sea salt, mineral dust and soil or anthropogenic sources: incomplete combustion of fossil/biofuel, wind-driven or traffic-related suspension of roads [1]. This type of dust can originate from gold mine tailings storage facilities and has become problematic for various communities that live nearby. This situation occurs in South Africa even though guidance for the management of dust disposition was created and established through the SANS1929:2005 dust-fall guideline and the National Dust Control Regulation of 2013. The fact that the country has such standards should have hypothetically solved any dust deposition problems South Africa might have experienced [2].

Windblown deposited dust is often a significant nuisance problem faced in South African urban and peri-urban areas due to the prevailing dry climatic conditions, extensive surface mining and mineral processing [3]. This dust often results in air pollution. The National Environmental Management Air Quality Act (NEM: AQA, 39 of 2004) defined air pollution as any change in the composition of the air caused by smoke, soot, dust (including fly ash), cinders, solid particles of any kind, gases, fumes, aerosols and odorous substances. This definition is similar to that of the World Health Organisation (WHO), which defined air pollution as a contamination of the indoor or outdoor environment by any chemical, physical or biological agent that modifies the natural characteristics of the atmosphere [4].

Gold-mining waste has been estimated as accounting for 221 million tons or 47% of all mineral waste produced in South Africa, making it the largest single source of waste and pollution [5]. There are approximately 270 tailings storage facilities in the Witwatersrand Basin, covering 400 km^2^ in surface area, which store this waste [6]. Tailings storage facilities are residue of the milling process used to extract valuable mined ores. Moreno et al. [7] defined tailings as the crushed, sand-like by-product refuse material which is generated during extraction, crushing, grinding and milling procedures of mined ore during the mining process. Emissions generated from mining activities are often associated with particulates, such as PM_10_, PM_2.5_ and dust fallout.

A number of TSFs are unlined and unrehabilitated, posing an environmental challenge, including water contamination by acid mine drainage (AMD), air pollution by windblown dust as well as particulate emissions [8]. In 2013, the West Rand District Environmental Management Framework estimated approximately 21.21 t/day particulate emissions from TSFs in the Merafong Municipality, the largest emitter in the West Witwatersrand Basin [9]. These impacts also include physical and aesthetic modification of the environment and challenges with sustainable vegetation cover as a result of soil contamination, since tailings contain toxic heavy metals.

Tailings storage facilities in the Witwatersrand area have, for many years, been a major source of air pollution due to fugitive dust emissions. Fugitive dust emissions from TSFs can affect the quality of life, health and well-being of neighbouring communities, for this study area evidence scientifically quantifying the negative impact has not been conducted except for a household survey in 2018. In the household survey, the residents perceived the dust from TSF6 to negatively affect their health and socio-economic status due to medical costs. In the Witwatersrand, dust fallout remains a prominent environmental hazard during the spring season, where wind erodible particulate matter (PM10) concentrations can reach up to 2000 µg/m^3^ [10,11,12,13,14].

A study by Oelofse et al. [15] noted that mine closure and an increase in AMD have critical consequences for mining-affected communities. This view is supported by Warhurst and Norhona [16], Claassen [17] and Ojelede et al. [14]. At the time of these studies, sudden/premature mine closure was uncommon. As a result, even to date, there are very few impact assessments studies linking sudden mine closure with dust deposition to environmental impacts. Most studies look at one aspect of the problem. The present study is the first of its kind to look at sudden mine closure (due to mine liquidation), linking it with dust deposition and its environmental impacts. Sudden mine closure occurs when a mine closes before its scheduled period [18]. A gold mine in the West Witwatersrand Basin was liquidated in the year 2013. Due to confidentiality agreements, the name of the mining company will not be mentioned in this paper. It is the perception of the surrounding community in this mine area that the unrehabilitated TSFs are responsible for air pollution by dust deposition.

The purpose of this research was to conduct an air quality impact assessment on the surrounding community of the gold mining village found in the West Witwatersrand Basin. This study investigates whether the community’s perceptions about the dust emanating from TSFs are valid and seeks to recommend possible solutions to address the dust problem. The study draws special attention to TSF6 which is the tailings storage facility the community had mostly complained about.

The focus of this study was to identify and critically assess the dust impacts during the period of mine liquidation. In order to achieve this research aim this study had the following research objectives; to conduct a particle size distribution analysis to investigate the health threats posed by the dust size fraction. To simulate the dust deposited from the surrounding tailings storage facilities in the vicinity of TSF6 and the gold mining village using the AERMOD dispersion model. To conduct a chemical analysis of all TSFs material surrounding the gold mining village, to investigate the impacts, potential toxicity and health threats that dust poses.

This study uses dispersion modelling to assess the impacts of dust deposition in the area and verify the validity of opinions about the threats posed by dust from the TSFs in the area. The result of this investigation were used to provide convincing solutions for the dust problem in the area. It appears that despite some studies being conducted on community complaints about unrehabilitated TSFs, a lack of support for communities through science persists when communities initiate litigation against a mining company. There is no convergence and integration between indigenous knowledge and factual evidence through science. This paper attempts to bridge the gap between lay knowledge and science as well as demonstrate the interaction between a liquidated mine, the natural environment and the society in which the mine is located.

### Background

**Figure 1:**
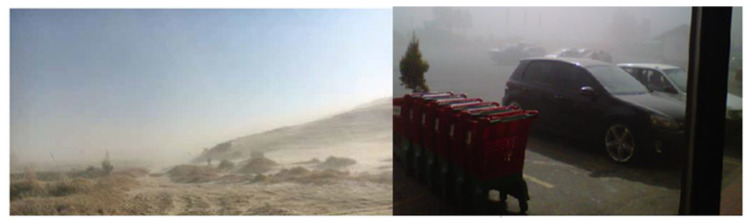
A windy day in the gold mine village [19].

Since the sudden mine closure occurred in 2013 due to a gold mining company being liquidated, the surrounding community has been complaining about dust. The gold mine village community, including the surrounding business owners, school and clinic, specifically complain during the windy season (August to October). In a household survey study conducted in 2018 by Mpanza and Moolla [19], the community stated that the dust triggers respiratory illnesses and community members are thus required to spend money to treat these illnesses. The residents of the gold mine village were asked in the household survey whether they considered dust to be a problem (environmental and socio-economic cost) to the community. The majority (94%) of the residents responded in the affirmative [19]. The residents further stated that the rehabilitation of TSFs discontinued when the sudden mine closure occurred. This marked the beginning of wind-blown dust being generated from TSFs. Hence, this paper, reports on the dust deposition impacts emanating from TSF6 and the surrounding tailings dumps. Figure 1 illustrates a windy day in the gold mine village, as observed by the surrounding affected community with dust emanating from TSF6.

## 2. Materials and Methods

### 2.1. Study Area

**Figure 2:**
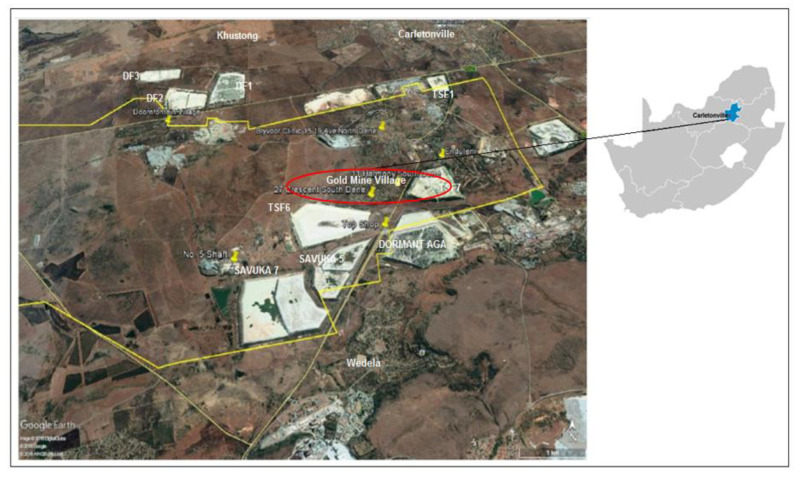
Study site with all TSFs in the vicinity of the gold mine village community.

The study area is classified as an urban environment located 6 km South West of Carletonville town. It involves all areas in the vicinity of TSF6, TSF1, TSF7, Dormant AGA, Doornfontein 1 (DF1), Doornfontein 2 (DF2), Savuka 5 and Savuka 7. The surrounding receptor communities include the gold mine village ward 5 and ward 27, Doornfontein mine village, Wedela, Carletonville and Khutsong. The Merafong Municipality which hosts the study site of this paper is estimated to have approximately 23 TSFs [20]. Figure 2 illustrates the tailings storage facilities assessed in this study.

### 2.2. Methodology

Various factors contribute to the dispersion, transformation and eventual removal of particles, from the atmosphere and the ground. Such factors include local meteorology, topography, land-use, source features (e.g., point, area, volume, line or pit source and source dimensions) and source strengths. A typical air quality assessment involves the assessment of measured ambient air quality data or dispersion modelling results. These are useful tools which evaluate the current state of air at a location, assessing air pollution and its sources at a number of locations. Total Suspended Solids (TSP) and PM10 are particulate matter investigated in this study as pollutants of concern. TSP and PM10 are investigated since they are particularly associated with mining activities. The US Environmental Protection Agency USEPA [21] stated that approximately 50% of the TSP is emitted as PM10, especially from mining sources.

The dispersion modelling conducted in this study is guided by the South African Regulations Regarding Air Dispersion Modelling of 2014. Reference to the British Columbia Air Quality Dispersion Modelling Guideline of 2015 and the Good Practice Guide for Atmospheric Dispersion Modelling, New Zealand of 2004 were used as best practice in the impact assessment. This study firstly considered emissions inventory for West Witwatersrand area, and reviewed the regulatory requirements and health thresholds for identified key pollutants. Dispersion modelling was conducted to determine the impacts on the receiving environment in the vicinity of the gold mine village and a screening assessment is carried out to determine compliance with the National Ambient Air Quality Standards (NAAQSs) and Dust Fall Control Regulations (NDCR).

**Figure 3:**
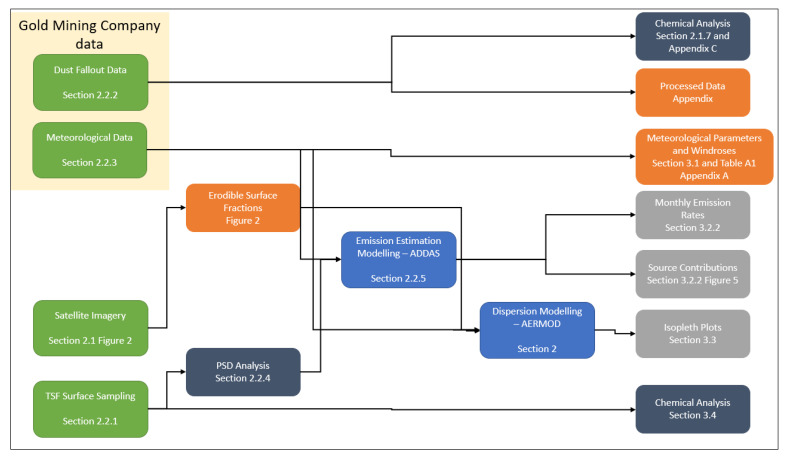
Process flow of the study methods.

The focus of this assessment was on the wind erodible sources, primarily the tailings storage facilities which, over time, have been partially vegetated. Other sources, such as the main mining roads and rock dumps, have not been included together with other sources such as crushing and screening and plant emissions. The main mining road is tarred and at the time this study was conducted, there was no active mining. Waste rock dumps next to TSF1 were not included as sources of windblown dust since they contribute negligible particulate emissions owing to the large particle size of the material. The vegetated portions of the TSFs were excluded from the assessment. The assumption is, negligible wind erosion occurs on the vegetated areas. A source apportionment was conducted on Google Earth by digitising the various TSFs to obtain the surface area. It should be noted that no significant changes were observed to the surface cover of the TSFs during the study period. Figure 3 summarises the methodology and processes followed in data collection and data analysis of this study.

To assess the effects of dust deposition and its impacts on the gold mine village community, the AERMOD dispersion model (AERMOD Version 09292, Lakes Environmental, Ontario, Canada,) was applied. The AERMOD was selected since it is a recommended model for sophisticated, near-source applications in all terrain types (where near-source is defined as less than 50 km from source). AERMOD models the TSFs as an area source. The community is located less than 100 m away from TSF6 and 200 m away from TSF7 which is far below the buffer distance recommended by the Chamber of Mines Guideline of 500 m. The model is commonly applied to Level 2 assessments; the gold mine village case is classified as a Level 2 assessment as per the Regulation for Air Dispersion Modelling of 2014. Apart from the gold mine village, other communities which might be affected by dust from surrounding TSFs include Carletonville town, Khutsong South and Wedela, located within a 50 km radius from the centre of TSF6 (see Figure 2). AERMOD is a Gaussian-plume type dispersion model which assumes a steady state meteorology and a fairly flat topography. The input parameters include, source data, meteorological data (pre-processed by AERMET model), terrain data and data on the nature of receptor grid. AERMOD has a range uncertainty of −50–200%; the accuracy improves with fairly strong wind speeds and neutral atmospheric conditions. The uncertainty comes as a result of modelling the physics, input errors and stochastic processes.

#### 2.2.1. Surface Sampling

Material samples were selected from eight TSFs. The samples were chosen due to their proximity to the community of interest (gold mine village). The TSFs assessed include Dormant AngloGold Ashanti, TSF1, TSF6, TSF7, Savuka 7, Savuka 5, Doornfontein 1, and Doornfontein 2. The selection of tailings included all TSFs which occur within a 10 km radius from the gold mine village. The village is shown within a red circle in Figure 2 and consists of ward 5 and ward 27.

The samples were scooped from the top centre surface, crest and slope surface of each tailings dam on all sides of the tailings. This material was mixed in one sample bag as one representative sample for each of the eight tailings storage facilities (a total eight representative samples were analysed). Material from the top centre of the TSF represents the core deposited material as original material. Side slope material represents material eroded from the top layer by wind and water. Chemical analysis and particle size distribution was conducted on this sample data. The particle size analysis from the surface material defines surface roughness, together with moisture content, clay content, silt content, particle density and bulk density, which are required inputs in the ADDAS model.

#### 2.2.2. Monitoring Data: Dust Fallout Sampling

**Figure A1:**
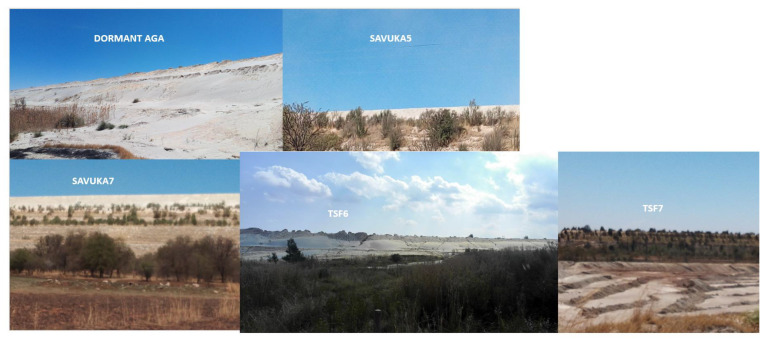
Tailings Storage Facilities sites.

The sampling of dust fallout was conducted in all the areas marked in yellow representing dust buckets (see Figure 2). Samples were collected from the dust fallout monitoring campaign which uses a method called the American Society for Testing and Materials Standard Method for Collection and Analysis of Dustfall (ASTM-1739). The single dust bucket collects dust fallout, PM_10_ and PM_2.5_ particulate as it lands in the bucket. The dust data was collected to validate the dispersion modelling results and to assess the particle sizes and chemical content of the actual dust particles. At the AngloGold Ashanti meteorological station (Mponeng Plant Station), meteorological data, dust fallout and PM10 has been monitored since 2012 using an airborne sampler. That data was supplied for this study. For each tailings storage facility, three dust samples were collected for chemical analysis and particle size distribution analysis. Each of these samples represented the duration of the windy season in 2018, from August to October (see Appendix A Figure A1).

#### 2.2.3. Meteorological Data

Meteorological characteristics affect the rate of emissions from fugitive dust sources and govern the dispersion potential and eventual removal of pollutants from the atmosphere [22]. To characterise the meteorological setting of the area, data from AngloGold Ashanti meteorological station (Mponeng Plant Station) was used, consisting of wind speed, precipitation, relative humidity and wind direction (see Appendix A Table A1). The data covered the period of mine liquidation, which began in 2012 and continued till 2017. The year 2012 marks the year just before the gold mining company was placed under liquidation in 2013. Hourly data of the aforementioned period was obtained and input files were generated using the AIRMET pre-processor for the dispersion simulations.

#### 2.2.4. Particle Size Distribution

To characterise each TSF, the particle size distribution, moisture content, clay content, silt content, particle density and bulk density were analysed as agents of particle entrainment, transport and deposition from the surface material. The particle size distribution was undertaken on the surface material using the Malvern Master Sizer system. Particle aerodynamic diameters determine if and for how long dust remains airborne, its likelihood of being inhaled and its site of deposition in the respiratory system.

#### 2.2.5. Emissions Quantification

Input files were prepared according to the Marticorena and Bergametti [23] model as part the windblown dust emissions quantification in the study area. The Marticorena and Bergametti [23] model accounts for variability in source erodibility by parameterising the erosion threshold (based on particle size distribution of the source) and roughness length of the surface material. The model also takes into account soil crusting related to friction velocity, these control the horizontal and vertical movements of dust. The emission rates were determined using the Airborne Dust Dispersion model from Area Sources (ADDAS) from the entire study site. This is an Airshed Planning Professionals’ in-house model [24]. The ADDAS model uses the threshold friction velocity of the particle size and the vertically integrated horizontal dust flux (see Equations (1)–(3)) based on the Marticorena and Bergametti [23] model and Burger [24]:

(1) $$E_{i}=G_{i}10^{\left(0.134C-6\right)}$$

(2) $$G_{i}=0.261\frac{\rho_{a}}{g}U_{i+1}^{3}\left(1+R_{i}\right)\left(1-R_{i}\right)$$

(3) $$R_{i}=\frac{U_{i+1}}{U}$$

where $E_{i}$ = emissions rate (size category); C = clay content (%); $\rho_{a}$ = air density; $G_{i}$ = gravitational acceleration, U = frictional velocity; and $U_{i+1}$ = threshold friction velocity (size category 1).

The ADDAS Model key inputs are summarised in Table 1. To cover the entire study area (as shown in Figure 2) a 7.5 km by 7.5 km receptor grid with a 100 m resolution was used for dispersion modelling purposes. According to the Greek National Pollutant Inventory [NPi] [25], an emission factor of 0.4 kg/ha/h should be adopted for TSP while a factor of 0.2 kg/ha/h should be used for PM10. These emission factor values are supported by Environment Australia 2001. The values were used as part of data validation, improving certainty in the modelling process. The US EPA finds that the friction velocity of 5.4 m/s initiates erosion of coal from a storage pile; this was used as a guide in this study [26]. Other scholars calculated a wind speed of 9 m/s as the speed required to initiate erosion from tailings storage facilities in New Brunswick and Ontario, Canada. Table 1 summarises all the data inputs for the ADDAS model [8].

The TSFs were considered to be inactive as no active mining was taking place in the study area at the time of data collection. The surface roughness length was considered to be the same for all tailings as the TSFs possess similar characteristics.

#### 2.2.6. Meteorological Data Analysis

**Figure A2:**
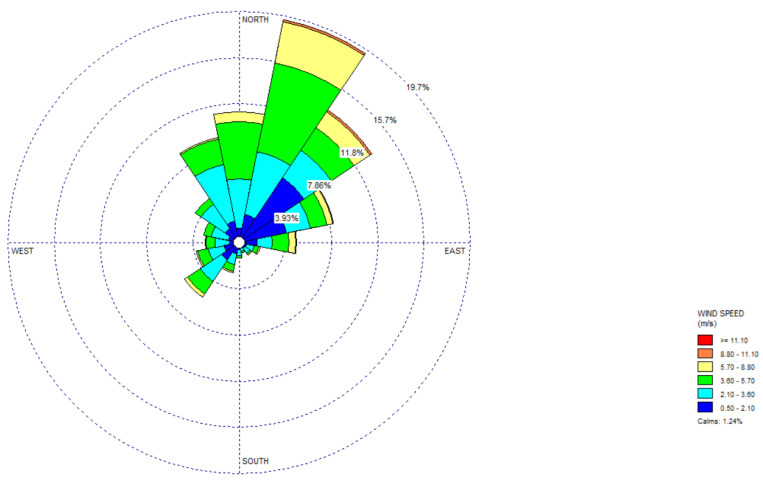
Wind Rose diagram of the study area.

The meteorological data was analysed through wind rose diagrams illustrating wind direction and wind speed (see Appendix A Figure A2). The meteorological data was used as input in the ADDAS model to determine the atmospheric dispersion potential in the study area.

#### 2.2.7. Chemical Analysis

To characterise contaminants within the selected TSFs, chemical analysis was conducted for all soil samples and dust samples collected in the area. The analysis included XRF and ICP-MS analysis. The ICP-MS analysis indicates the toxic metals which have potential to affect human health. The ICP-MS analysis followed the USEPA 3051a procedure. The X-Ray Fluorescence (XRF) analysis was also undertaken. This is a non-destructive technique used to determine elemental composition of the sampled material.

#### 2.2.8. Model Validation and Limitations

All measured dust fallout data was used to compare with the dispersion simulations (see Appendix A and Table A2). The simulations only include emissions associated and in close proximity of the gold mine village ward 5 and ward 27. The vegetated area and rock dumps were excluded from the assessment as they were assumed not to contribute to the windblown dust owing the immovable large particle size material of the dumps. The study relied on aerial photographs of the area as part of source data characterisation. The TSFs were the only source considered in the modelling process and therefore other sources adding to the ground level concentrations are not factored in the modelling.

## 3. Results

### 3.1. Meteorological Parameters

The extent to which pollution accumulated or dispersed in the atmosphere (atmospheric dispersion potential) depends on meteorological factors such as wind speed, wind direction, air temperature etc. The wind speed determines the distance of downwind transport and the rate of dilution as a result of plume stretching [27]. The wind speed also governs the mechanical turbulence affected by surface roughness. Wind direction determines the pathway that the pollutants will follow and the spread of the winds [22,28,29]. Air temperature determines plume buoyancy, mixing and inversion layers. Rainfall represents the removal of pollutants from the atmosphere. Atmospheric stability determines the heating of the ground and mechanical mixing due to the friction effects from the earth’s surface.

The average wind speed is 2.94 m/s in this area. The strongest wind speeds are greater than 6 m/s, which occurred mostly during the spring months. The USEPA [26] suggests that a wind speed threshold of 5.4 m/s is typical for tailings storage facilities to initiate wind erosion. The general wind direction in the study area is Northerly and North-Easterly (see Wind Rose in Appendix A Figure A2). July to August represents the driest months, with low precipitation. This allows wind erosion to occur with ease. The average rainfall during the liquidation period was 67 mm/month. The study area has neutral atmospheric conditions in general. The wind speed assisted in calculating the friction velocity required by the ADDAS model. All meteorological parameters are summarised in Appendix A Table A1.

### 3.2. Dust Emission Rates

#### 3.2.1. Particle Size Distribution

**Figure 4:**
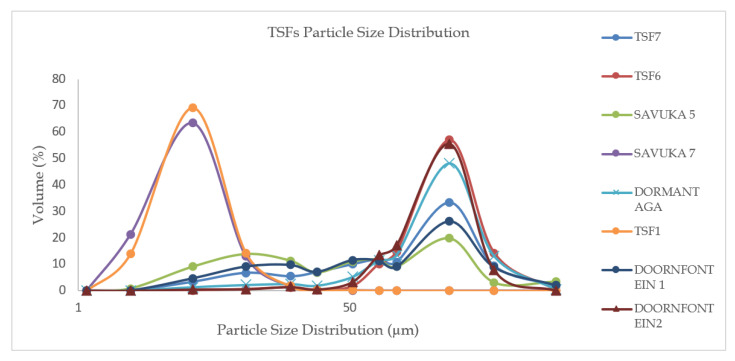
Particle size distribution for study site tailings storage facilities.

**Figure 5:**
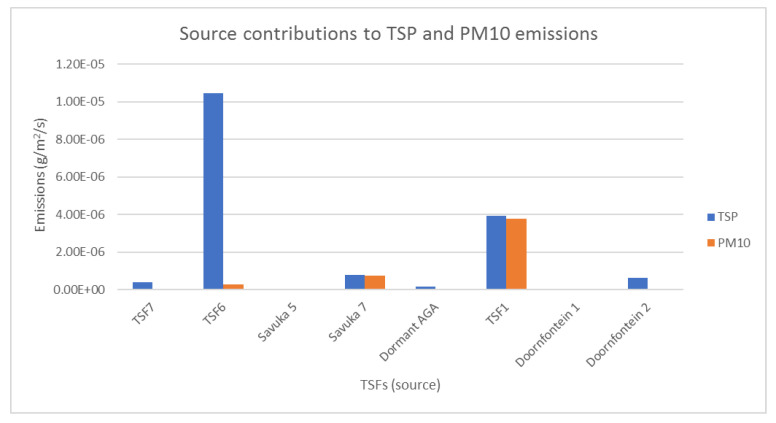
Source contribution to overall emission rates TSP and PM10.

As shown in Figure 4, TSF1 has the bulk (97%) of its particle size distribution ranging between <2 and 30 µm (fine material), followed by Savuka 7. From the graph, it is evident that TSF6 and Doornfontein 2 have primarily coarse material, while TSF7, Savuka 5, Dormant AGA and Doornfontein 1 show a mix of both fine and coarse material. It is expected that TSF1 will show high contributions of PM10 while TSF6 will show the highest dust fallout and TSP emissions (see Figure 4 and Figure 5). TSF1 and Savuka 7 are the biggest health threats, owing to its high respirable fraction of particulate matter. These two TSFs surround the gold mine village, although TSF1 is located 800 m away from the first line of houses in the gold mine village.

Tegen and Fung [30] state that global dust emissions consist of 13% of particle size ranging from 0.5–1 µm, while 65% in the range of 1–35 µm and 22% in the range of 35–50 µm. In this study, approximately 60% of the particle size is in the inhalable and respirable dust fraction range. From the size analysis, it is clear that the community in the gold mine village is affected by PM10 and dust fall owing to its location close to TSF1 and TSF6. Fine particulate matter is known to induce subtle health effects, mostly respiratory diseases and physiological potency [31,32,33].

**Figure A3:**
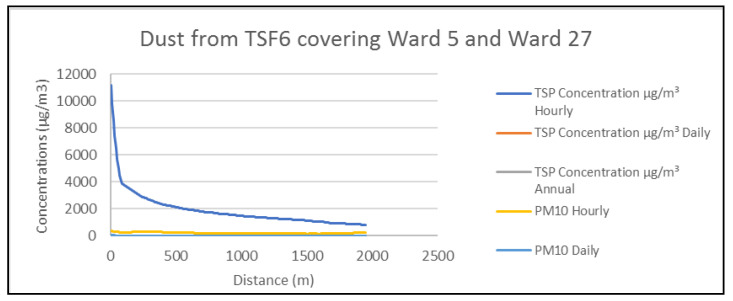
Distance covered by dust in the mine village.

It appears that TSF1 underwent hyperfine milling of ore during reprocessing and this resulted in fine material. TSF6 has chiefly coarse particle sizes; this storage facility has not been reprocessed to date. For the community close to the gold mine, TSF6 contributes nuisance dust (inhalable dust) covering a distance of 60–2000 m (see Appendix B Figure A3) which triggers community complaints, while TSF1 consists mainly of the respirable dust fractions. According to Ojelede et al. [13], inhalable particulate matter is available on normal and windy days near TSFs.

#### 3.2.2. PM 10 and TSP Emission Rates

TSF 6 contributes the highest TSP emissions and has the largest surface area (1,086,773 m^2^) while TSF1 contributes the highest PM10 emissions, as illustrated in Figure 5. This is no surprise as this TSF showed the highest percentage of particle size distribution in the <2–30 µm range and is partially vegetated. It can be deduced from Figure 5 that the impact of TSF6 is mainly nuisance dust based on the representatives samples analysed.

**Figure 6:**
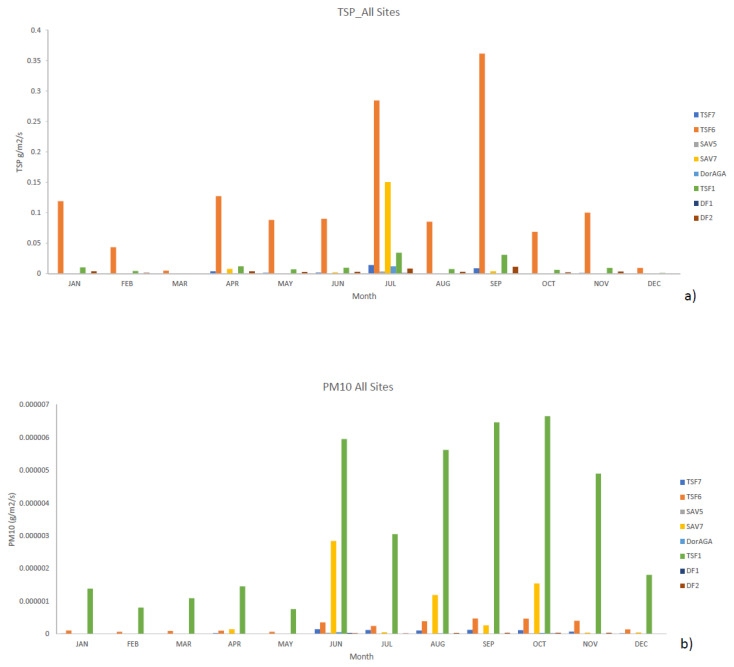
Seasonal emission rates (**a**) TSP and (**b**) PM10.

During the spring season (August–October), the highest emission rates were calculated for TSP in TSF6 in 2012, 2013 and 2016 (see Figure 6). In all these years, the gold mining company in close proximity to the community was under the supervision of the liquidator. Similarly, for PM_10_ the highest emission rates were reached during the windy season (June–October). The highest PM_10_ rates were calculated at TSF1 in 2014 and 2016; and at Savuka 7 (SAV7) in 2016. The better vegetated TSFs such as Savuka 5 (SAV5), Dormant AGA (Dor AGA) and TSF7 showed the least emission rates. This reinforces the premise that rehabilitated TSFs are a lower environmental and human health threat. The high emission rates of TSP and PM_10_ during the windy season are consistent with the surrounding communities’ high complaints around this time.

### 3.3. Dispersion Simulations

**Figure 7:**
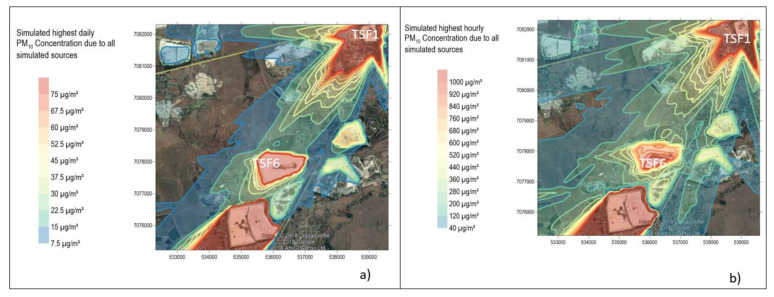
Simulated highest daily PM10 (**a**) and highest hourly PM10 (**b**) concentration due to all simulated sources.

**Figure 8:**
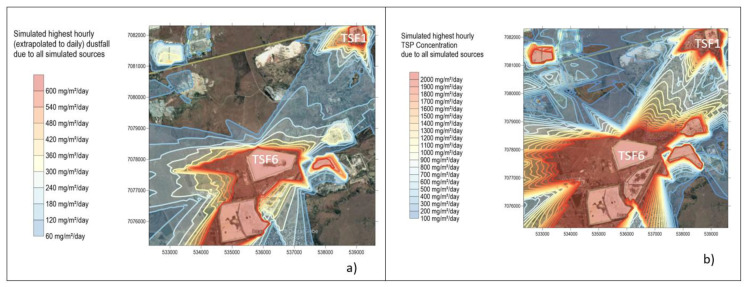
(**a**) Simulated highest hourly Dustfall and (**b**) TSP due to all simulated sources.

**Figure 9:**
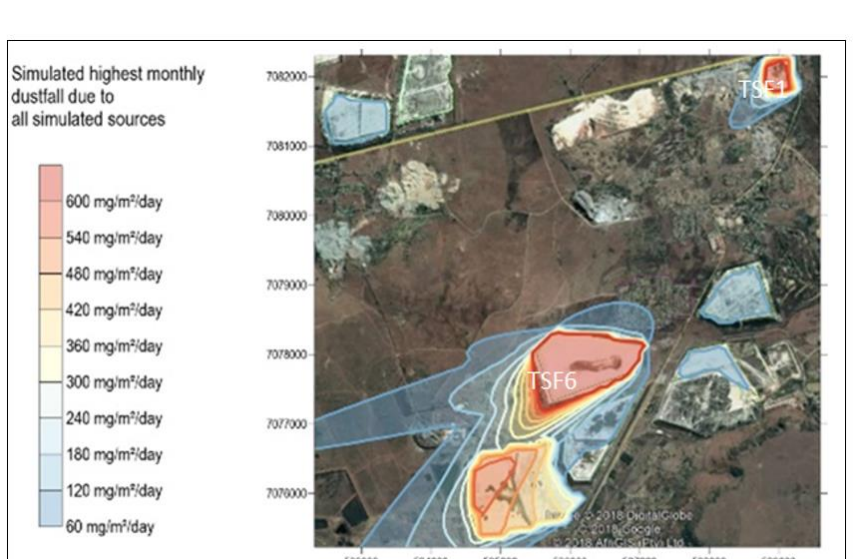
Simulated highest monthly dust fall due to all simulated sources.

**Figure 10:**
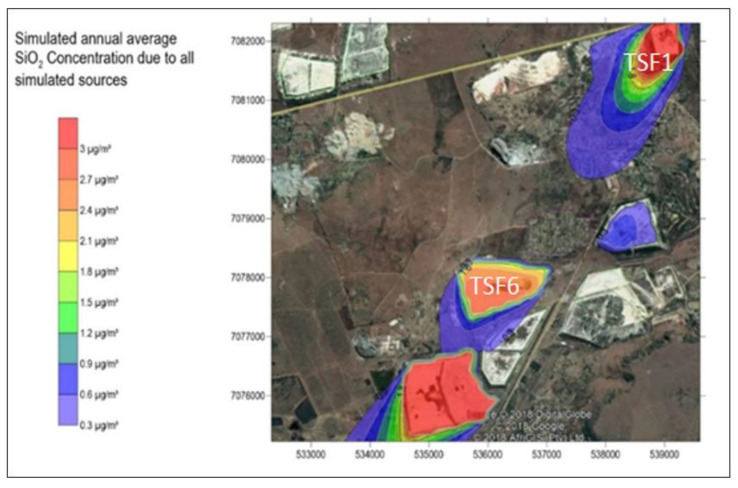
Simulated annual average SiO_2_ concentration due to all simulated sources.

The model simulation results are the ground level concentrations (GLCs) in µg/m^3^ for PM10 and dust deposition rate in mg/m^2^/day for dust fall and TSP. The results are shown as graphical presentations of isopleths shown from Figure 7, Figure 8, Figure 9 and Figure 10. The isopleth plots depict interpolated values from the concentrations simulated by AERMOD for each identified receptor grid point. The hourly, daily, monthly and annual averages are shown for worst cases and these were compared with the NAAQS and NDCR as shown in the Appendix A, Appendix B and Appendix C.

#### 3.3.1. PM10 Simulations

No clear exceedance of the South African air quality standard of 40 µg/m^3^ annual average was evident due to the incremental impact of the evaluated TSFs only. In TSF1, located 800 m from the first line of houses in the gold mine village, PM10 GLCs reached up to 32 µg/m^3^ annual average which is within the standard (see Appendix B Table A3). On an annual basis there were no significant changes observed in PM10 GLCs average.

TSF6 and TSF1 seem to be directly impacting the community with no clear exceedances of the 75 µg/m^3^ daily air quality standard. Figure 7b summarises the highest hourly ground level concentrations of PM10 in the study area. It should be noted that there is a possibility that even though a high hourly average concentration is simulated at certain locations (TSF1, TSF6 and Savuka 7), this may have been a possibility for one or two hours at a time while the 24-h average concentrations may have remained in compliance with the standard. The community of Wedela (located south of Savuka 7) seems to be the most affected as it is located downwind of all the TSFs assessed in this study area. This community is outside of the study area thus not shown in Figure 7 and is not investigated further. There is a significant threat of impacts in the short term which could be the source of the community’s complaints on specific days and months. Although simulated concentrations appear to be below the daily standard, the short-term exposure is enough of a threat to trigger respiratory diseases. Several studies suggest that short-term exposure to particulate matter is associated with negative health effects, even at low concentrations of exposure [34]. Furthermore, short-term exposure to outdoor air pollution PM_10_ and PM_2.5_ can worsen respiratory symptoms. Pope and Dockery [34] studied daily mortality in relation to PM_10_ in Utah (USA) and found that daily average of 365 µg/m^3^ had recorded effects on mortality. Short-term exposure to PM_10_ is associated with lower respiratory symptoms, medication use and small reductions in lung function [35]. The community of this gold mine village made mention of having to buy medication to treat respiratory diseases during the windy season [19]. Particulate matter is known to exacerbate asthma due to cellular oxidative stress, initiated by particle-produced free radicals. There is strong evidence suggesting that short-term increase in ambient concentrations of PM_10_ is associated with increase in PM_10_-related morbidity [36].

In this study, it should be noted that while the cumulative impacts from the TSFs together with additional background concentrations were not evaluated, the highest short term impacts at the gold mine village are expected to occur as a result of wind erosion from the TSFs during high wind speed episodes. The incremental impact from the TSFs only is in compliance with the NAAQS.

It is an interesting finding to observe that the community perceives TSF6 as a major health threat whereas this study shows based on the bulk sampling analysed that the impact of TSF6 is mainly nuisance dust. The gold mine village is situated on the downwind side of TSF1 in terms of the prevailing wind direction, while TSF6 poses a significant nuisance to the surrounding community and the nearby shopping centre on the downwind side of this TSF. This study suggests that when a mining operation is closed suddenly due to liquidation, poorly rehabilitated TSFs can generate dust posing health threats and a nuisance.

#### 3.3.2. TSP and Dust Fall Simulations

Simulated highest hourly dust fallout and TSP are shown in Figure 8. The TSP has implications on dust fall as it is coarse-grained and is eventually deposited.

**Figure A4:**
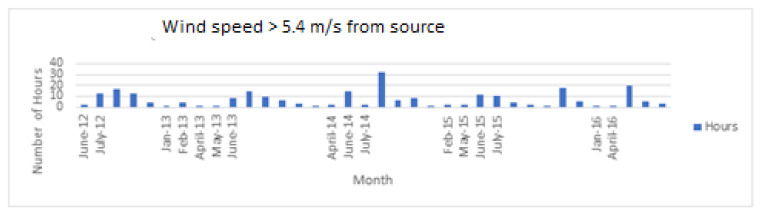
Wind speed > 5.4 m/s from the SW Sector.

The simulated highest hourly dust fall and TSP dust deposition rates from all sources show high short-term dust fall which is equivalent to daily dust fall rates higher than 600 mg/m^2^/day. The highly affected receptors appear to be located away from the gold mine village to the South West (see Figure 8a,b respectively). The highest simulated dust fallout rates is 600 mg/m^2^/day, from TSF1, TSF6 and Savuka 7. The highest hourly dust fallout rates simulations are very high for both dust fallout and TSP. This is another indication of high exposure in the short term. During the entire study period in August, a total of 30 h (highest hours) were recorded of dust fallout, TSP and particulate matter at high wind speeds >5.4 m/s (see Appendix B Figure A4) for the entire study period. Research finds that air pollution in cities of developing countries is responsible for some 50 million cases per year of chronic coughing in children younger than 14 years of age [37]. Chay and Greenstone [38] find that higher concentrations of total suspended particulates (TSPs) are strongly associated with higher rates of infant mortality. Furthermore maternal exposure to pollution also raises infant mortality.

There are exceedances of the NDCR with dust fall rates reaching 600 mg/m^2^/day at TSF1, TSF6 and Savuka 7. These exceedances, however, have no clear impact on the community of the gold mine village. The NDCR allows for two days non-sequential exceedances per year (see Appendix B Table A4).

#### 3.3.3. Simulated Results and Measured Data

Simulations and measured data were expected to differ, as simulations only included emissions associated with the TSFs in the vicinity of the gold mine village area as modelled. The sampled or measured dust fall rates include sources from areas close to the mine village (see Figure 2). Table A2 in Appendix A shows the measured and the simulated results. It is evident that simulated dust fall rates are lower than the measured dust fall rates. This was expected due to the assumptions made and the modelling inputs being only from TSFs. A major reason for a mismatch in the results could be that the parameters in the model are not readily amenable to reflect local factors, thus leading to discrepancies in outputs. The AERMOD model is originally set up for the United States of America environment and not the South African conditions.

### 3.4. TSFs Chemistry

The elemental investigation aimed to find out whether there was any silica content in the dust, which is a potential health threat to the surrounding communities. The study finds that silica was the most abundant of all the other minerals in all the TSFs, ranging from 65–93%. Other major elements included Al, K, Fe, Mg, and Mn in small quantities. The ICP-MS results indicated the presence of As, Pb, U, Cr, Ni, Cd, Au and Se. Makgae [39] and Maseki [40] note that numerous mining residential areas are at risk of high silica and radioactivity contamination. This is due to the silica and uranium content found in the tailings storage facilities, especially in the Witwatersrand Basin.

To examine the potential health impacts that could be posed by the heavy metals, enrichment factors were calculated. According to Dudu et al. [41], the enrichment factor (EF) method is one way of quantifying the anthropogenic pollution of a given site. The assumption made in calculating EF is that the ratio is 1 for elements not above crustal average. EF greater than 2 shows enriched elements above crustal average, meaning additional sources have contributed to the elemental composition. In this study Au, U and As have a high enrichment factor, significantly above crustal average, ranging from 72–359, 30–82 and 33–317 respectively. TSF7, TSF6, TSF1 and Dormant AGA were assessed as they closely surround the gold mine village community.

The elemental content of the TSFs is a product of the ore and materials used in the gold treatment and extraction processes [42,43]. The major concerns, however, are silica and uranium, which are both carcinogenic at high levels over a period of time. These are known to pose respiratory diseases and cancer.

It is expected to observe this trend as it was found by Maseki et al. [8] that As, Pb, U and Au are highly enriched in the West Witwatersrand Basin. This is owing to the increased uranium and gold content of the Dominion Reef mined in the Basin. Studies conducted in the Witwatersrand Basin investigated airborne radioactivity levels through radiometric surveys and confirmed high doses of uranium in and around TSFs [44].

A high silica content is also recorded from the TSFs. Acute exposure to high concentrations of silica can cause cough, shortness of breath and pulmonary alveolar lipoproteinosis (acute silicosis), provided it is fresh cut. After chronic but lower workplace exposure to silica for 6 to 16 years, the small airways become obstructed, as measured by pulmonary function tests [45].

The simulated annual average SiO2 concentration due to all simulated sources is shown in Figure 10. The Californian Office of Environmental Health Hazard Assessment provides a chronic inhalation reference exposure level of 3 µg/m³ for respirable crystalline silica. The simulated annual average of SiO2 is shown to exceed 3 µg/m³ at TSF1, TSF6 and Savuka 7. TSF1 presents a threat to the community at ward 5, as wind is blowing from this source 2 km towards the community, however, it appears not to reach ward 27. TSF6 and Savuka 7 have effects on the Wedela community as it is downwind of these tailings dumps.

According to the Mine Health and Safety Act, [9] Section 11.6 if silica content in coal dust is greater than 5%, employers must establish and maintain a system of medical surveillance. Section 11.7 mentions that medical surveillance must be established and maintained for employees working in places in excess of 10% or 0.1 mg/m^3^ Occupational Exposure Limit (OEL) for crystalline silica dust. Respirable crystalline silica is considered a great concern and a greater danger than ordinary dust [46,47]. Epidemiological studies have established correlations between respirable crystalline silica particulate matter with acute and chronic respiratory disorders, for example, chronic silicosis [45,46,47,48,49,50]. No silica exposure standards exist for the public; only occupational standards exist in South Africa.

Makgae [39] and Maseki [40] note that numerous mining residential areas are at risk of high radioactivity contamination. This is due the uranium content found in the tailings storage facilities, especially in the Witwatersrand Basin. To examine the potential health impacts that could be posed by the heavy metals shown in Table A5 in the Appendix C, enrichment factors [EF] are calculated. Toxic metals such as As, Pb, U, Zn, Ni, Cr and Cd were considered when calculating enrichment factors. The equation for calculating the EF is as follows:

(4) $$EF=\frac{M_{x}\times Fe_{b}}{M_{b}\times Fe_{x}}$$

where $M_{x}$ and $Fe_{x}$ are the concentrations of element M and Fe in the sample x and $M_{b}$ and $Fe_{b}$ are the mean concentrations of the element M and Fe in the continental crust [51].

Iron is used as a reference element (43,200 ppm), as suggested by Taylor and McLennan [52]. In this study, Au, U and As have an extremely high enrichment factor, far greater than crustal average, ranging from 72–359; 30–82 and 33–317 respectively. Similar results were documented by Maseki et al. [8] that Cd, Au and As have high enrichment factors, falling in the class of extremely high enrichment (EF > 40). Gold enrichment is not a great concern since it is biologically inert, therefore poses no potential harm to human health [53]. Ohlander et al. [54] explain the enrichment of metals (As and Cd) in pyrite rich tailings by adsorption to pyrite or iron-oxyhydroxides occurring with the oxidation of pyrite. The enrichment of Au and As has similarities with previous work in the Witwatersrand, where the association between As and Au was attested [55]. According to Simon et al. [56] the association between As and Au is justified by the fact that As plays a major role in enhancing the adsorption of gold complexes pyrite surfaces. It is also noticed in this study that TSF6 has significantly lower levels of U238 than TSF1 at values of 3.77 ppb and 12.25 ppb respectively.

## 4. Discussion

From the size analysis, it is clear that the community in the gold mine village is affected by PM_10_ and dust fall owing to its location close to TSF1 and TSF6. Fine particulate matter is known to induce subtle health effects, mostly respiratory diseases and physiological potency [32,33]. The elemental investigation aimed to find out whether there was any silica content in the dust, which is a potential health threat to the surrounding communities. The study finds that silica was the most abundant of all the other minerals in all the TSFs, ranging from 65–93% (see Appendix C Table A6). Other major elements included Al, K, Fe, Mg, and Mn in small quantities. The ICP-MS results indicated the presence of As, Pb, U, Cr, Ni, Cd, Au and Se. Furthermore, the chemical analysis showed that Au, U, and As were extremely highly enriched, with EF > 40. Of all the tailings, TSF6 has the lowest uranium content, thus posing a minimal contribution to radioactvity. This is possibly due to the mining processes used during production. TSF1 is a potential health threat in the long term while TSF6 is a nuisance in the short term. The community members of the gold mine village were possibly complaining about the nuisance dust in the short term at high wind speeds >5.4 m/s. Tailngs storage facilities with discontinued rehabilitation are in fact a source of wind-blown dust in the windy season especially when a mine is closed suddenly due to mine liquidation. The dust from TSFs affects surrounding communities based on its characteristics, posing a health threat (TSF1) or a nuisance (TSF6) in this study area. The dispersion modelling conducted in this study, based only on the TSFs source appeared to have underestimated the GLCs and the dust deposition rate. It is anticipated that, should all sources be taken into account the values for GLCs and deposition rate would appear much larger and be comparable to the measured values.

Due to the size of the population in the residential area, there are higher chances of adverse human health effects occurring. To show the potential effects of the dust to human health in the community of the gold mine village, a full health risk assessment should be conducted by a qualified toxicologist or a relevant health scientist. Epidemiology studies should be conducted on the population affected by the dust storms so that the actual effects can be quantified. The determination of the actual effects will assist in guiding responsible authorities on the appropriate remedies that can be implemented as well as the medical interventions that can be given to the affected residents.

This study therefore suggests that when a mining operation is closed suddenly due to liquidation, poor rehabilitation of TFSs can generate windblown dust, posing nuisance and health threats. The results indicate exposure risks to dust fallout and PM_10_ with high silica content from the TSFs. The results emphasise the need to ensure that TSFs are rehabilitated so that they do not pose a threat to the community’s health and quality of life. The results have also indicated that Savuka 5, Dormant AGA and TSF7 that have been rehabilitated and are less of an environmental and human health threat. These results further emphasise the need to rehabilitate TSFs in order to mitigate the environmental threats posed to the nearby community members.

## 5. Conclusions

The community provided perspectives on their daily experiences of the environment. The dispersion model provides the overall scientific evidence about the status of the environment with respect to air quality. In the 21st century, the integration of indigenous knowledge and science cannot be overlooked, especially to provide fast monitoring and management of the environment. The community perspectives, in terms of short term dust impacts, were comparable to the dispersion simulations and the emission rate calculations. The dust emissions are prevalent during the windy season, with August–September being the highest months, as correctly pointed out by the community. Based on the particle size analysis of the representative samples it appears the community, was inaccurate in saying that TSF6 is a major health threat. It is, in fact, a nuisance in the windy season. In the entire study period (2012–2017), only 30 h showed high wind speeds capable of transporting dust over large distances greater than 6 km. An analysis of the source material and dust samples showed the presence of particles in the inhalable and respirable range in certain TSFs, which are known to be more toxic once inhaled. In the short term, the modelled PM10 has potential to trigger respiratory diseases. Modelled PM showed that the daily PM10 concentrations at TSF6, TSF1 and Savuka 7 were slightly above the acceptable exposure limit. The results from the assessment revealed that strong Northerly and Easterly winds blowing to the South West are more frequent compared to other wind patterns and lead to tailings dust deposition south of the TSFs. It is recommended that during a sudden mine closure period due to mine liquidation, the Department of Mineral Resources and Energy exercise their authority to ensure that liquidators rehabilitate TSFs on behalf of a mining company. This can ensure management of TSFs while mitigating environmental and health impacts. Despite the establishment of a single environmental system’s concern with promoting cooperative government amongst the national departments responsible for mineral resources and environmental affairs the impact of the single system on the original legislative and executive powers does not seem to have been properly considered. It is recommended that in the single environmental system, alignment of mining, company and environmental laws be included to close gaps creating synergies to eliminate poor environmental remediation during and post mining activities.

There is also a need for contributions from all stakeholders during the process of policy making and development to ensure that there are no loopholes in mining and environmental management policies. The stakeholders include the government, companies, and the civil society.

Community adaption training for all people in the residential areas that receive high dust emissions from TSFs is also recommended. Since this study also aimed at bridging the gap between scientific and lay knowledge, community members need to be equipped with the scientific knowledge that has been shown in this study. Such training will assist in equipping residents with the knowledge and skills in monitoring and minimising the adverse effects of dust on their health. Furthermore, community education can provide knowledge and understanding with regards to dust, thus managing community complaints.

Though it is suggested in the study that the community complaints and high dust episodes are comparable during the windy season; in future studies a complaints register from the community needs to be kept, to qualify the dust data showing the exact days of high wind episodes. A correlation between days of high complaints and high dust would prove beneficial for such a study.

Specific guidelines for mine closure are provided under Section 2 of the National Environmental Management Act (NEMA). These guidelines require the mining right holder to rehabilitate the environment, set aside a financial provision for such rehabilitation, and to retain liability for environmental damage even after closure of the operation. Despite having these regulations in place, there is still a high degree of non-compliance in the mining industry. The fact that some TSFs have not been rehabilitated means that the gold mining company did not fully comply with the regulations during operation. There is therefore a need for law enforcers to ensure compliance in order to protect the community’s rights to a clean and healthy environment. Continued non-compliance with laws and standards will keep on exposing the lives of community members to health hazards and this may result in an increased burden on the already stressed health system in the country. This calls for long-term closure and post-closure management measures that are aimed at protecting the environment and the health of communities in gold mining areas.

## Figures and Tables

**Table 1:** ADDAS model key inputs for dispersion modelling, moisture content and surface cover.

Location	Density (kg/m^3^)	Moisture (%)	Surface Cover (%)	Undisturbed-Non-Vegetation (Y/N)	Min U_*t_	Erodible Fraction (%)
TSF7	2640	0.69	48	N	5.4	52
TSF6	2640	0.09	10	Y	5.4	90
SAVUKA 5	2640	4.14	19	N	5.4	81
SAVUKA 7	2640	0.48	27	N	5.4	73
DORMANT AGA	2000	0.07	87	N	5.4	13
TSF1	2000	0.04	20	Y	5.4	80
DOORFONTEIN 1	2000	0.07	90	N	5.4	10
DOORFONTEIN 2	2000	0.08	1	N	5.4	99

## Appendix A

**Table A1:** Meteorological parameters from the WW Mponeng Plant Station.

Parameter Period from January 2012 to December 2017	
Wind Direction	N; NE
Wind Speed	2.94 m/s
Precipitation	67 mm/month
Relative Humidity	54–71%
Atmospheric Stability	Fairly neutral
Solar Radiation	283
Receptor Grid	7.5 km × 7.5 km
Temperature	10–23 °C

Wind Rose

**Table A2:** Modelled and Measured dust fall, highest monthly dust deposition at all receptors.

Location Name	Distance from TSF6 (m)	Highest Monthly Modelled Dust Fall Rate (mg/m^2^/day)	Highest Monthly Measured Dust Fall Rate (mg/m^2^/day)	Measured Dust Deposition Annual Average (mg/m^2^/day)
No. 5 Shaft	1408	68	450	193
27 Crescent South Dene	300	96	750	215
Doornfontein Village House	3984	10	480	200
1 Harmony South Dene	866	23	490	168
Top Shop	400	45	600	277
Joseph’s house	2500	14	540	190
Clinic	2315	24	500	184

## Appendix B

**Table A3:** National Ambient Air Quality Standard for Particulate Matter (PM_10_).

Averaging Period	Concentration	Frequency of Exceedance	Compliance Date
24 h	120 µg/m^3^	4	Immediate-31 December 2014
24 h	75 µg/m^3^	4	1 January 2015
1 year	50 µg/m^3^	0	Immediate-31 December 2014
1 year	40 µg/m^3^	0	1 January 2015

**Table A4:** National Dust Control Regulation.

Restriction Areas	Dustfall Rate (mg/m^2^/day, 30 Days Average)	Permitted Frequency of Exceeding Dust Fall Rate
Residential area	D < 600	Two within a year, not sequential months.
Non-residential area	600 < D < 1200	Two within a year, not sequential months.

## Appendix C

**Table A5:** ICP-MS results for tailings storage facilities.

Composition	K 39 ppb	Cr 52 ppb	Mn 55 ppb	Fe 57 ppb	Ni 60 ppb	Zn 66 ppb	As ppb	U 238 ppb	Se ppb	Cd 11 ppb	Au 197 ppb	Pb 208 ppb
**Location**												
TSF7	375.22	49.43	687.56	14,737.49	194.58	182.23	32.15	50.50	1.47	0.53	0.49	7.55
TSF6	241.43	51.18	126.28	14,865.82	28.85	21.16	30.06	3.77	0.31	0.03	0.10	4.40
TSF1	298.66	45.95	77.45	11,906.95	23.70	37.57	18.24	12.25	0.24	0.03	0.26	4.60
DORMANT AGA	283.03	59.96	162.83	17,229.47	45.07	30.68	24.09	22.00	0.69	0.07	0.27	10.86
SAVUKA 7	923.90	129.19	1110.43	29,434.75	75.34	71.53	30.01	67.28	0.55	0.13	0.35	27.35
SAVUKA 5	3644.52	75.84	210.75	20,048.29	70.84	54.12	29.86	76.074	0.48	0.18	0.46	21.86
DOORFONTEIN 1	359.15	70.28	164.24	15,235.13	51.66	43.42	18.37	4.05	0.37	0.07	0.06	5.54
DOORNFONTEIN 2	197.51	14.62	14.54	3605.72	6.126	6.29	8.11	6.84	0.60	0.04	0.11	2.55

**Table A6:** XRF results for tailings storage facilities.

Composition	SO_3_%	TiO_2_%	Al2O_3_%	CaO%	Fe_2_O_3_%	K_2_O%	MgO%	MnO%	Na_2_O%	P_2_O_5_%	SiO_2_%
**Location**											
TSF7	0.27	0.30	5.70	0.41	2.86	0.65	0.76	0.06	0.15	0.06	85.32
TSF6	-	0.26	4.61	0.24	2.23	0.59	0.58	-	0.08	-	90.37
TSF1	-	0.32	5.22	0.21	2.25	0.64	0.55	-	0.09	-	89.00
DORMANT AGA	-	0.44	5.61	0.49	3.47	0.65	0.96	-	0.22	0.05	86.44
SAVUKA 7	0.62	0.43	7.07	0.90	4.26	0.90	1.02	0.12	0.27	0.05	80.08
SAVUKA 5	5.37	0.35	6.48	1.24	3.56	1.40	3.76	-	3.11	0.06	65.94
DOORFONTEIN 1	0.14	0.38	7.88	0.55	3.78	1.05	1.47	-	0.16	-	81.60
DOORNFONTEIN 2	-	0.24	3.60	-	1.20	0.38	0.12	-	-	-	93.29
